# Possible Receptor Mechanisms Underlying Cannabidiol Effects on Addictive-like Behaviors in Experimental Animals

**DOI:** 10.3390/ijms22010134

**Published:** 2020-12-24

**Authors:** Ewa Galaj, Zheng-Xiong Xi

**Affiliations:** Addiction Biology Unit, Molecular Targets and Medications Discovery Branch, Intramural Research Program, National Institute on Drug Abuse, Baltimore, MD 21224, USA; ewa.galaj@nih.gov

**Keywords:** cannabidiol, cocaine, CB1 receptor, CB2 receptor, TRPV1, 5-TH, addiction

## Abstract

Substance use disorder (SUD) is a serious public health problem worldwide for which available treatments show limited effectiveness. Since the legalization of cannabis and the approval of cannabidiol (CBD) by the US Food and Drug Administration, therapeutic potential of CBD for the treatment of SUDs and other diseases has been widely explored. In this mini-review article, we first review the history and evidence supporting CBD as a potential pharmacotherapeutic. We then focus on recent progress in preclinical research regarding the pharmacological efficacy of CBD and the underlying receptor mechanisms on addictive-like behavior. Growing evidence indicates that CBD has therapeutic potential in reducing drug reward, as assessed in intravenous drug self-administration, conditioned place preference and intracranial brain-stimulation reward paradigms. In addition, CBD is effective in reducing relapse in experimental animals. Both in vivo and in vitro receptor mechanism studies indicate that CBD may act as a negative allosteric modulator of type 1 cannabinoid (CB1) receptor and an agonist of type 2 cannabinoid (CB2), transient receptor potential vanilloid 1 (TRPV1), and serotonin 5-HT_1A_ receptors. Through these multiple-receptor mechanisms, CBD is believed to modulate brain dopamine in response to drugs of abuse, leading to attenuation of drug-taking and drug-seeking behavior. While these findings suggest that CBD is a promising therapeutic candidate, further investigation is required to verify its safety, pharmacological efficacy and the underlying receptor mechanisms in both experimental animals and humans.

## 1. The History of Medicinal Cannabidiol

The history of *Cannabis sativa* (cannabis) reaches to ancient Asia, where the plant was cultivated for religious, medicinal or textile purposes [1,2]. The first medicinal use of cannabis goes back to 4000 BC and relates to the treatment of pain, constipation, menstrual cramps and malaria [3,4]. In the beginning of the Christian Era, cannabis was used together with wine as an analgesic during surgical procedures [1]. The therapeutic use of cannabis was introduced to the Western medicine in the nineteenth century and served as analgesic, anti-inflammatory, anticonvulsant, antiemetic, anesthetic, antitussive, and appetite stimulant [2]. There were also early anecdotal reports that cannabis can alleviate anxiety, depression, mania and other psychological conditions. Despite the apparent therapeutic effects of cannabis, its use in Western medicine decreased significantly in the twentieth century. This decrease was due to several factors, including the discovery of vaccines, more efficacious medications, concerns over cannabis’ psychoactive properties and its increasing recreational use [2].

During the rise of modern medicine, cannabis was not recognized among the medical community because of a lack of reliable scientific evidence supporting its efficacy. There was anecdotal evidence that cannabis produced therapeutic effects; however, initial attempts to validate the therapeutic effects of cannabis often fell short. This was due to different strains of cannabis and methods of preparation being used in the studies, making it difficult to compare findings across studies and draw comprehensive conclusions. In addition, newly introduced legislation (e.g., the Marijuana Tax Law of 1937, the Controlled Substances Act of 1971) restricted the use of cannabis for medicinal, recreational and experimental purposes [5]. Under these new laws, cannabis was classified as a Schedule I controlled substance, bringing its medicinal use and academic research to a virtual halt.

Despite restrictive registration, the interest in the recreational use of cannabis intensified in the 1960s and 1970s, and scientists were able to isolate its psychoactive and therapeutic constituents [6,7], leading to a new scientific interest in cannabis and its medicinal use. In early 1960s, the Mechoulam lab first isolated and described the structure of cannabidiol (known as CBD) and ∆^9^-tetrahydrocannabinol (∆^9^-THC) allowing scientists to study their psychoactive and therapeutic effects [6]. In the late 1960s, the Mechoulam group began testing isolated cannabinoids in primates and discovered that ∆^9^-THC, but not CBD, causes sedative effects [8]. In 1980, Dr. Mechoulam and colleagues published the results of the clinical trial showing that individuals with severe epilepsy experienced improved conditions after CBD treatment without experiencing any side effects [9]. Unfortunately, despite this breakthrough discovery, this publication was largely ignored among the medical and scientific communities. Some of the reasons pertain to the stigma surrounding cannabis and psychedelics since the 1960s and 1970s.

In 2013 the story of Charlotte Figi surfaced, the little girl who had suffered over 300 grand mal seizures per week, with no medication able to prevent the episodes or reduce their intensity [10]. CBD was reported to eliminate her seizures, saving her life. The story gained national attention and galvanized support for CBD legislation as a medical treatment. In 2014, the Farm Bill (i.e., the Agriculture Act of 2014) was signed into law, legalizing the cultivation of cannabis containing <0.3% of ∆^9^-THC at the state level. Soon some states passed legislation for the legalization of medical CBD, and in 2018, the US Food and Drug Administration (FDA) recognized and approved Epidiolex, the drug containing CBD, for the treatment of seizures associated with pediatric Lennox-Gastaut syndrome or Dravet syndrome, making a significant milestone in modern medicine [11]. The Farm Bill of 2018 legalized the cultivation and sale of hemp at the federal level and officially removed it from the Controlled Substances Act, Schedule I, making research and medicinal development of CBD more accessible.

In the last decade, CBD has gained popularity in the scientific community and its efficacy has been screened for a variety of medical and psychological conditions. The literature provides evidence supporting CBD’s therapeutic utility in the treatment of neuropathic pain, epilepsy, inflammation, bacterial infections, nausea, loss of appetite and sleeplessness [12,13,14,15]. CBD has been also shown to be effective for neuropsychiatric disorders including substance use disorders (SUDs) [16,17,18,19,20]. There is a number of articles that have reviewed recent research regarding CBD actions against psychostimulants [17,20], opioids [21,22], ∆^9^-THC [23] and alcohol [24,25] in both humans and experimental animals. However, the majority of them focus on experimental evidence supporting or refuting the use of CBD for the treatment of SUDs, while little attention has been given to recent research regarding the possible receptor mechanisms underlying CBD’s action in different animal models of drug abuse and addiction. In this mini-review article, we first briefly review the rationale and major historical events supporting the development of medicinal CBD. We then review more recent findings regarding the pharmacological effects of CBD against opioids, psychostimulants and alcohol, and the involvement of potential receptor mechanisms.

## 2. CBD Attenuates Opioid Addictive-Like Behaviors, Possibly through CB1 and 5-HT_1A_ Receptor Mechanisms

Systematic research on the efficacy of CBD on drug addiction did not begin until 1975 when Hine and colleagues published a series of studies demonstrating that CBD can potentiate the pharmacological effects of ∆^9^-THC to attenuate the physical signs of morphine withdrawal in rats [26,27]. Hine et al. found no evidence that CBD alone can attenuate withdrawal symptoms in rats [27], but a year later, another report was published demonstrating that CBD reduces naloxone-precipitated withdrawal in mice with a history of morphine exposure [28]. These early studies opened the doors to further exploration of the therapeutic utility of CBD in the treatment of SUDs. In 2009, Hurd’s group investigated the effects of CBD treatment on heroin self-administration and relapse in rats. Systemic injections of CBD (5 and 20 mg/kg) were reported to have no effect on heroin self-administration or drug-primed reinstatement of heroin-seeking [29]. However, CBD was effective in reducing cue-induced reinstatement of heroin-seeking 24 hours and 2 weeks following administration, suggesting that CBD’s effect on heroin-seeking is protracted [29].

In a conditioned place preference (CPP) paradigm, when mice were pretreated with CBD prior to a morphine conditioning session, CBD blocked the development of morphine CPP [30], suggesting that CBD has the ability to block the rewarding effects of opioids and can serve as a preventive therapeutic. When administered prior to the CPP test, CBD reduced the expression of morphine CPP in rats [31]. In addition, CBD administered right after the reactivation trials disrupted reconsolidation of drug-reward memory as indicated by the absence of the preference during the drug- or stress-induced reinstatement [31], suggesting that CBD has the ability to attenuate memories associated with opioid reward and consequently to reduce risks of relapse. In rats with established morphine CPP, CBD blocked conditioned place aversion precipitated by naloxone in the morphine context, suggesting that CBD also has the ability to attenuate the severity of withdrawal symptoms in rats [31].

However, little is known about the mechanisms through which CBD inhibits opioid reward-associated learning and memories, cue-induced heroin-seeking and opioid withdrawal. Ren and colleagues reported that cue-induced heroin-seeking is associated with alterations in the expression of AMPA (α-amino-3-hydroxy-5-methyl-4-isoxazolepropionic acid) GluR1 and the CB1 receptor in the nucleus accumbens (NAc), and such alterations were normalized after the CBD treatment, suggesting that some of CBD’s effects against heroin may be mediated by the AMPA and CB1 receptor mechanisms [29]. In addition, other studies showed that CBD reduces the rewarding effects of morphine in an electrical intracranial self-stimulation (ICSS) paradigm and this effect is mediated by serotonin 5-HT_1A_ receptors [32]. Specifically, intra-dorsal raphe infusions of WAY100635, a selective 5-HT_1A_ antagonist, blocked the attenuating effects of CBD, suggesting that CBD interferes with opioids’ action through a 5-HT_1A_ receptor mechanism.

## 3. CBD Attenuates Cocaine Addictive-Like Behavior by the CB1, CB2, 5-HT_1A_ and TRPV1 Receptor Mechanisms 

The first empirical studies assessing the efficacy of CBD against cocaine were published in the 1990s. CBD was shown to protect mice against cocaine- or norcocaine-induced liver hepatotoxicity [33,34]. It was two decades later when the therapeutic potential of CBD for cocaine-driven behaviors was evaluated in a systematic way. In the first such study, conducted by Mahmud and colleagues, rats trained to self-administer cocaine under a fixed interval of 20 s (FI-20 s) and progressive ratio (PR) schedules of reinforcement were treated acutely with CBD either 30 min or 24 h prior to the test. CBD (5 and 10 mg/kg) failed to attenuate cocaine self-administration under both schedules and during cue-induced reinstatement [35]. However, other follow-up studies indicated that higher doses of CBD (15–40 mg/kg) are required to reduce cocaine self-administration [36,37]. Repeated administration of 20 mg/kg CBD was able to reduce cocaine taking in mice over time [36]. In our recent report, we found that CBD, at 20 and 40 mg/kg, failed to reduce cocaine self-administration maintained by 0.5 mg/kg/infusion under fixed ratio 1 (FR1) and PR schedules of reinforcement in rats [37]. However, when the cocaine dose was lowered to 0.25 mg/kg/infusion, CDB significantly attenuated PR response for cocaine. In a self-administration paradigm with multiple cocaine doses that changed within each session, CBD at 20 mg/kg was effective against the 0.03, 0.06 and 0.12 mg/kg doses of cocaine, but not 0.25 and 0.5 mg/kg [37].

In regards to other cocaine-related behaviors, CBD has been shown to block the acquisition of cocaine place preference (CPP) when administered prior to conditioning sessions [36]. In addition, CBD can facilitate the extinction of cocaine- and amphetamine-induced CPP [38]. In rats previously trained for cocaine CPP, CBD disrupted the reconsolidation of cocaine memories and preference when CBD was administered immediately after drug cue exposure [31]. In a brain-stimulation reward (BSR) paradigm, CBD (20 mg/kg) was shown to block the reward-enhancing effects of cocaine [37] but to have no effect at lower CBD doses [32]. CBD also failed to alter cocaine sensitization when administered alone [36] or in combination with ∆^9^-THC [39]. However, in a reinstatement test, transdermal application of CBD for 7 days was shown to be effective in reducing cocaine-seeking in rats with a history of self-administration and this effect persisted for a long time even when the CBD treatment was terminated [40]. Together, these data suggest that CBD has certain therapeutic effects in attenuation of cocaine reward and relapse to drug-seeking behavior.

The receptor mechanisms underlying the above CBD action remain unclear. Early in vitro ligand binding assays indicate that CBD has low (μM range) binding affinities to CB1 or CB2 receptor orthosteric binding sites (Table 1). However, more recent receptor binding and multiple intracellular signal functional assays indicate that CBD has unexpectedly high binding affinity and potency (with nM of K_i_, EC_50_ or IC_50_ values) as a CB1R negative allosteric modulator, CB2 receptor antagonist/inverse agonist or partial agonist, and TRPV1 and GPR55 agonist (Table 1). Based on these new findings, we have recently explored the roles of CB1, CB2, GPR55, TRPV1, 5-HT_1A_, and mu opioid receptor (MOR) in CBD’s action in multiple animal models related to drug abuse and addiction (Table 2). We found that CB1, CB2, 5-HT_1A_ and TRPV1 receptors are critically involved in CBD action in cocaine self-administration, sucrose self-administration, and cocaine-enhanced BSR. In contrast, we did not find evidence supporting the involvement of GPR55 and MOR in CBD’s action in cocaine self-administration [37].

### 3.1. CB1R Mechanism

**Figure 1:**
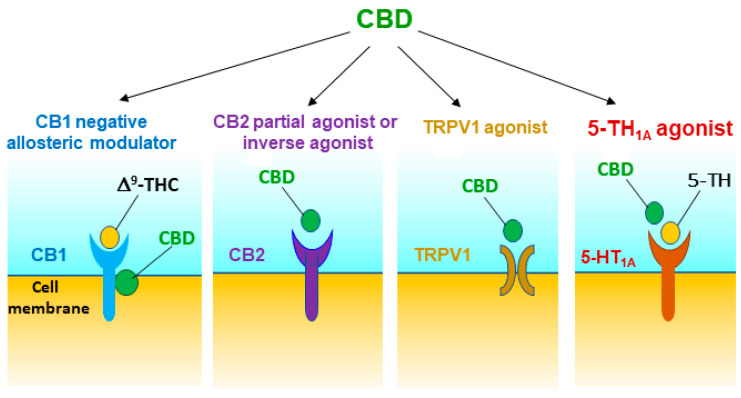
Potential receptor mechanisms underlying CBD’s action against drug reward and addiction. The results from in vitro receptor binding and functional intracellular signalling assays and in vivo behavioral studies with pharmacological and transgenic approaches suggest that CBD may act as a CB1R negative allosteric modulator, a CB2R partial agonist or antagonist/inverse agonist, and a TRPV1 and 5-HT_1A_ receptor agonist. Multiple receptor mechanisms together produce protective and therapeutic effects against drug abuse and addiction.

Although CBD has low affinity for the CB1R orthosteric binding site [46], it displays high nM affinity for the CB1R allosteric binding site and functionally act as a negative allosteric modulator (Table 1, Figure 1) [41,42,43,51]. CBD pretreatment was reported to be able to reduce the affinity of orthosteric ligands to CB1Rs and CB1R-dependent intracellular signaling such as ∆^9^-THC- and 2-AG-induced CB1R internalization, β-arrestin recruitment and phospholipase C β3- and ERK1/2-phosphorylation [41,42,43,51]. To determine the role of CB1R in CBD’s action in experimental animals, we trained mice for oral sucrose self-administration. We found that systemic administration of CBD (10, 20, and 40 mg/kg, i.p.) produced a dose-dependent reduction in sucrose self-administration in rats and in wild-type (WT) mice [52]. Unexpectedly, CBD was more efficacious in transgenic CB1 receptor-knockout (CB1-KO) mice than in WT mice. Similarly, pretreatment with AM251 (a selective CB1R antagonist) potentiated CBD-induced reduction in sucrose self-administration, suggesting that CB1R antagonism may underlie the additive or synergistic effects of CBD on sucrose self-administration [52]. However, not all evidence supports this finding. Pretreatment with AM251 failed to enhance CBD’s action in cocaine self-administration or electrical BSR in rats [37], suggesting that the CB1R antagonism produced by CBD on the allosteric binding sites is not sufficient in the attenuation of cocaine reward. This is consistent with literature reports that blockade of CB1Rs by AM251 was able to inhibit cocaine self-administration in some reports [37,53,54], but not in others [55,56,57,58,59]. In addition, repeated treatment with CBD increases the expression of CB1Rs in the striatum [36], suggesting possible interactions between CBD and CB1Rs.

### 3.2. CB2R Mechanism

In addition, CBD was reported to act as a CB2R antagonist or inverse agonist [41,49], a CB2R agonist or partial agonist [42,60], or a negative allosteric modulator of CB2Rs with nM binding affinity [44] (Table 1, Figure 1). To further determine the role of CB2R in CBD’s action in experimental animals, we used both pharmacological and transgenic approaches. We found that pretreatment with AM630 (a selective CB2R antagonist) prevented CBD’s attenuating effects on cocaine self-administration and cocaine-enhanced BSR in rats [37], suggesting that CB2Rs might be involved in CBD’s action and CBD itself may act as a functional CB2R agonist. Similarly, pharmacological blockade or genetic deletion of CB2R in CB2-KO mice blocked CBD-induced reduction in oral sucrose self-administration [52]. Systemic administration of JWH133 (a selective CB2R agonist) also produced a dose-dependent reduction in sucrose self-administration in WT but not in CB2-KO mice. Pretreatment with AM630 blocked JWH133-induced reduction in sucrose self-administration. These findings are consistent with previous reports that CB2R antagonism attenuates CBD-induced reductions in food intake, body weight and obesity [61,62,63], and attenuates CBD-produced neuroprotection [64] (Table 2).

**Table 2:** Receptor mechanism studies in vivo in reward-related behaviors in experimental animals.

Receptor Mechanism	Major Findings	References
CB1	CBD blocked CB1R up-regulation in rats after extinction from heroin self-administration	[29]
	Repeated treatment with CBD increased CB1R expression in the striatum	[36]
	Deletion of CB1R potentiated CBD-induced reduction in sucrose self-administration in CB1-KO mice	[52]
	AM251 failed to alter CBD-induced reduction in cocaine self-administration in rats	[37]
	SR141716A failed to alter CBD-potentiated extinction of cocaine- or amphetamine-induced CPP in rats	[38]
	AM251 failed to alter CBD-induced reduction in 75 mg/kg of cocaine-induced seizure	[65]
	AM251 blocked CBD-induced reduction in aggressive behavior caused by social isolation in mice	[66]
CB2	Deletion of CB2R blocked CBD-induced reduction in sucrose self-administration	[52]
	AM630 attenuated CBD-induced reduction in cocaine self-administration	[37]
	AM630 attenuated CBD-induced reduction in food intake, body weight and obesity	[67,68]
	AM630 failed to alter high doses (75 mg/kg) of cocaine-induced seizure in mice	[65]
TRPV1	TRPV1 antagonist blocks CBD-induced reduction in cocaine self-administration in rats	[37]
	TRPV1 deletion attenuates CBD-induced reduction in electrical shock-induced seizure in mice	[69]
5-HT_1A_	The 5-HT_1A_ antagonist, WAY100635, blocked CBD’s action in cocaine self-administration in rats	[37]
	WAY100635 attenuated CBD’s action on morphine-enhanced brain stimulation reward in rats	[32]
	Deletion of 5-HT_1A_ in the DRN abolished cocaine-induced CPP. 5-HT_1A_ antagonism in the DRN inhibited cocaine self-administration in rats	[70]
	Intra-NAc CBD inhibited spontaneous DA neuronal firing, which can be reversed by WAY100635	[71]
	WAY100635 attenuated CBD’s action in alcohol consumption in rats	[72]
	WAY100635 attenuated CBD-induced reduction in aggressive behavior caused by social isolation	[66]
GPR55	GPR55 antagonism did not alter CBD’s action in cocaine self-administration in rats	[37]
	GPR55 deletion failed to alter CBD’s action in cocaine self-administration in mice	
MOR & DOR	Naloxone had no effect on CBD action in cocaine self-administration	[37]

These findings are important, as growing evidence indicates that CB2Rs are expressed in the brain and are functionally involved in drug reward and addiction [73,74]. Particularly, CB2Rs have been found on midbrain dopamine (DA) neurons in the ventral tegmental area (VTA) [67,68] and their projection terminals in the NAc [75,76]. Therefore, we have recently proposed that a DA-dependent mechanism may underlie CBD’s attenuating effects on cocaine reward via a combination of multiple receptor mechanisms, including the CB2R mechanism [37,52]. This hypothesis is supported by our findings that pretreatment with CBD significantly attenuates cocaine-induced increases in extracellular NAc DA [37] and that intra-NAc microinjections of CBD inhibit VTA DA neuronal activity and attenuates amphetamine-induced hyperlocomotion [77]. Importantly, CBD alone does not significantly alter extracellular DA levels in the NAc [37]. This may, in part, explain why CBD itself is neither rewarding nor aversive.

### 3.3. 5-HT_1A_ Mechanism

In addition to CBD binding to the CB1 and CB2 receptors, Russo et al. [48] were the first to report that CBD also acts as a 5HT_1A_ receptor agonist. Since then, a number of studies indicated the involvement of 5-HT_1A_ receptors in CBD’s action in vivo, primarily its anxiolytic effects [70]. We have recently reported that pretreatment with WAY100135, a selective 5-HT_1A_ antagonist, was able to block CBD-induced reductions in cocaine self-administration and in cocaine-enhanced BSR, supporting an important role of 5-HT_1A_ in CBD’s action [37] (Table 2). In addition, intra-NAc administration of CBD also significantly inhibits spontaneous mesolimbic DA neuronal activity and burst firing, which can be reversed by 5-HT_1A_ antagonism [71], suggesting that the activation of 5-HT_1A_ receptors by CBD may modulate the mesolimbic DA system, producing the reward-attenuating effects described above (Figure 1).

### 3.4. TRPV1 Mechanism

It was reported that that CBD may also activate TRPV1 (also known as the capsaicin receptor) [78,79]. Therefore, we have recently examined this possibility. We found that pretreatment with capsazepine, a selective TRPV1 antagonist, was able to block CBD-induced reduction in cocaine self-administration and BSR [37], suggesting that TRPV1 activation may also contribute to the therapeutic effects of CBD (Figure 1). The precise TRPV1 mechanisms involved in CBD’s action in cocaine reward are unclear. As TRPV1 channels are found on glutamatergic neurons in the frontal cortex and on striatal GABAergic neurons [80], one possibility is that CBD may alter brain glutamate and GABA release via TRPV1, leading to a reduction in cocaine reward. Another possibility is that CBD may inhibit fatty acid-binding proteins (FABPs) that mediate anandamide (AEA) transport to its catabolic enzyme fatty acid amide hydrolase (FAAH), leading to an increase in extracellular anandamide (AEA) levels [46,47,81], which subsequently activate both CB1Rs and TRPV1 [82,83], altering cocaine-taking and cocaine-seeking behavior. We should note that CBD displays low affinity to FABPs and low efficacy in inhibiting AEA update [47]. CBD inhibits AEA hydrolysis only at very high concentrations (≥50 μM) in HeLa cells expressing rat, but not human, FAAH [47], suggesting that this action may play a limited role in CBD’s action in addiction-related behavior.

It is unknown how such multiple receptor mechanisms mediates CBD-attenuated effects on cocaine reward. Since CBD displays different binding affinities to different receptors (Table 1), one possibility is that it produces therapeutic effects through different receptor mechanisms at different drug doses. Another possibility is that the receptors discussed above may form functional heteromers or interact at intracellular signal molecule levels if they are co-expressed in a specific phenotype of neurons. Thus, CBD’s action at one receptor may alter another receptor’s signaling and function.

## 4. CBD Inhibits Methamphetamine Reward and Relapse by DA-Related Mechanisms

Methamphetamine (METH) is another widely abused and highly addictive psychostimulant. It has been reported that CBD at high doses (80 mg/kg, but not 20 or 40 mg/kg) can attenuate response to meth under a PR schedule of reinforcement, suggesting that CBD has the ability to reduce motivation and METH reward [84]. CBD can also reduce drug-primed reinstatement of METH-seeking in rats, suggesting its therapeutic potential in relapse prevention [84]. Intracranial injections of CBD directly into the NAc can block hyperlocomotion, stereotypy and behavioral sensitization induced by amphetamine [77]. CBD has been also shown to facilitate the extinction of amphetamine CPP [38] and to prevent meth-induced reinstatement of CPP in rats. Intracranial microinjections of CBD into the lateral ventricles attenuated the reinstated preference for meth-related environments under stress conditions (i.e., sleep deprivation) [85].

To date, specific receptor mechanisms underlying CBD’s action against METH addiction-related behaviors have not been reported. Evidence has shown that intra-NAc CBD can inhibit amphetamine-induced hyperlocomotion and behavioral sensitization by regulating downstream phosphorylation of the mTOR/p70S6 kinase signaling pathway within the NAc shell [77]. In addition, rats conditioned with METH show strong CPP associated with upregulation of the Sigma1 receptor and a number of intracellular molecules (such as AKT, p-AKT, GSK-3β, p-GSK-3β, CREB and p-CREB) in the prefrontal cortex (PFC), VTA, NAc and hippocampus. CBD attenuated METH-induced CPP in a dose-dependent manner by regulating the Sigma1R, p-AKT, p-GSK3β and p-CREB pathways across these brain regions [86]. These important findings suggest that CBD might have therapeutic potential on METH-driven behaviors and can reverse some of the METH-induced neuroplastic changes.

Furthermore, stress- or drug-induced reinstatement of METH CPP has been associated with the upregulation of a number of cytokines in the PFC and hippocampus, including interleukin-1β (IL-1β), interleukin-6 (IL-6), interleukin-10 (IL-10) and tumor necrosis factor α (TNF-α) [87]. Interestingly, CBD prevented the upregulation of IL-1β, IL-6 and IL-10 mRNA in the PFC, and also TNF-α, IL-1β and IL-6 mRNA in the hippocampus in rats undergoing drug-induced reinstatement. These cytokines are known to modulate the neuronal activity of monoamine neurons (e.g., DA neurons) indirectly by the release of neuroactive molecules from glia cells and directly by activating cytokine receptors located on DA and other monoamine neurons [88,89,90,91]. Thus, it has been proposed that METH re-exposure enhances the expression of pro-inflammatory cytokines such as IL-1β and TNF-α, leading to the release of neurotransmitters that are involved in the reinstatement of METH. CBD appears to attenuate this form of METH-induced neuroplasticity in the mesocorticolimbic DA system [87].

## 5. CBD Attenuates Alcohol Taking Possibly by the CB1, CB2 and 5-HT_1A_ Receptor Mechanisms

CBD also shows therapeutic potential for the treatment of alcohol use disorder. In a two-bottle paradigm, CBD (60 and 120 mg/kg, i.p.) reduced alcohol consumption and preference in mice. In addition, CBD was effective in reducing oral self-administration of alcohol, relapse and alcohol-induced hypothermia [92]. Males appear to be more sensitive to CBD than females, as acute treatment with CBD (30, 60, 90 mg/kg) attenuated alcohol intake in male mice, but in female mice, 90 mg/kg CBD was required to inhibit alcohol intake [93]. Interestingly, CBD was found to be effective in reducing context-and stress-induced reinstatement of alcohol-seeking, up to 138 days post-CBD treatment, even though brain CBD levels remained detectable only for 3 days post-treatment [40]. CBD also reduced anxiety and impulsivity in rats with a history of alcohol consumption [40]. However, CBD appears to be ineffective in reducing alcohol-induced locomotor sensitization in mice, unless combined with ∆^9^-THC (1:1 ratio) [94], suggesting that ∆^9^-THC can potentiate CBD’s action against alcohol.

The receptor mechanisms through which CBD inhibits alcohol intake remain unclear. After chronic CBD administration mice showed a significant reduction of tyrosine hydroxylase (TH) mRNA expression in the VTA and of *Oprm1*, CB1 and GPR55 in the NAc [92,93]. However, a significant increase in CB2 mRNA was found in the NAc [92]. In addition, a combination of CBD with naloxone can also reduce alcohol consumption and the associated gene expression of *Oprm1* in the NAc, TH in the VTA and 5-HT_1A_ in the dorsal raphe nucleus [72]. Interestingly, the administration of WAY100635 (a 5-HT_1A_ antagonist) significantly blocked the action of CBD + naltrexone. Although a causal relationship was not determined in these studies, these findings suggest that the attenuating effects of CBD on alcohol consumption and relapse might be mediated by mechanisms related to CB1, CB2, GPR55, MOR and 5-HT_1A_ receptors within the mesolimbic system.

In summary, growing evidence indicates that CBD could be a promising candidate for the treatment of substance use disorders. In experimental animals, CBD displays pharmacological efficacy in attenuation of drug reward and propensity to relapse. The receptor mechanisms underlying CBD action are very complex and are involved in multiple receptors, as reviewed here (Table 2). Pharmacological and transgenic studies indicate that CB1, CB2, TRPV1 and 5-HT_1A_ receptors are critically involved in CBD acts on in vivo. Blockade or genetic deletion of CB1Rs tends to enhance, while blockade or deletion of CB2R attenuated cocaine or sucrose self-administration, suggesting that CBD may act as an allosteric CB1R antagonist or a CB2R antagonist/inverse agonist or partial agonist (Figure 1). Similarly, pharmacological blockade of TRPV1 or 5-HT_1A_ receptors also attenuates CBD’s action in multiple behavioral paradigms of reward and addiction, suggesting that CBD may functionally act as a TRPV1 and 5-HT_1A_ agonist (Figure 1). As stated above, these receptors may functionally modulate VTA DA neuron activity and DA release in the NAc either directly or indirectly [73,95]. Thus, the mesolimbic DA system could act as one of the final common targets underlying CBD’s anti-addiction effects. Among the other targets such as GPR55, PPARγ, TRPA1, TRPM8, TRPV2, adenosine A_1_ and A_2A_, mu and delta opioid receptors and α1 glycine receptors [50,96,97,98], CBD displays very low affinity to these receptors with the half maximal effective concentration (EC_50_) or half maximal inhibitory concentration (IC_50_) values of 1–20 μM, suggesting limited roles of these receptors in CBD’s action. There is a lack of behavioral evidence indicating that GPR55 and MORs are involved in CBD action in animal models of addiction [37]. We note that CBD displays high binding affinity to DA D_2_ receptors (with a Ki value of 11 nM for D_2_ high affinity binding sites) and functionally acts as a partial D_2_R agonist; however, the functional role of D_2_ receptors in CBD’s action has not been explored in vivo. Given that clinical doses (800–1000 mg/day) of CBD are sufficient to occupy the functional D_2_High sites and produce antipsychotic effects [45], it is suggested that the D_2_R partial agonist action of CBD may contribute to the therapeutic effects against drugs of abuse. Together, all these findings are promising, while further preclinical studies and future clinical trials are necessary to fully evaluate the safety and efficacy of CBD and the underlying receptor mechanisms in the treatment of substance use disorders.

## Figures and Tables

**Table 1:** Cannabidiol (CBD) binding profiles in in vitro binding assays for the receptors or functional molecules involved in drug abuse and addiction.

Target	Function	CBD Action	References
CB1	Antagonist	Low affinity to CB1Rs in [^3^H]CP55940 binding assay (K_i_ = 3.3~4.9 μM); ↓ CP55940-enhanced GTPγS binding (K_B_ = 79 nM); ↓ CP55940-induced inhibition of cAMP formation (K_B_ = 545 nM); ↓ CP55940-induced β-arrestin recruitmrnt in CB1-expressing HEK-CRE cells (K_B_ = 547 nM)	[41,42]
	NAM	↓ Δ^9^-THC- or 2-AG efficacy and potency on arrestin2, PLCβ3 and ERK1/2 signaling in CB1-expressing HEK 293A cells (IC_50_ = 0.27~0.96 μM)	[43]
CB2	Antagonist	Low affinity to CB2Rs in [^3^H]CP55940 or [^3^H]WIN55212-2 binding assays (CBD-K_i_ = 4.2 μM vs. ∆^9^-THC-K_i_ = 3.3 μM) ↓ CP55940-enahnced GTPγS binding in CB2-expressing CHO cells (K_B_ = 65 nM) ↓ CP55940-induced inhibition of cAMP formation (K_B_ = 641 nM); ↓ CP55940-induced β-arrestin recruitmrnt in CB2-expressing HEK-CRE cells (K_B_ = 420 nM);	[41,42,44,45]
	NAM	↓ JWH133-induced reduction in forskolin-stimulated cAMP formation (IC_50_ = 3 nM) ↓ JWH133-induced ERK1/2 phosphorylation in HEK cells expressed hCB2 (IC_50_ = 29 nM)	[44]
	Inverse agonist	↓ GTPγS binding in CB2-expressing CHO cells (EC_50_ = 503 nM)	[41]
	Partial agonist	Unexpectedly high affinity to CB2Rs in [^3^H]CP55940 binding assays (CBD-K_i_ = 34 nM vs. ∆^9^-THC-K_i_ = 15 nM) ↑ BRET response, ↑ intracellular CRE activity in CRE and BRET assays in CB2-expressing HEK cells	[42]
GPR55	Antagonist	↓ CP55940-enhanced GTPγS binding in GPR55-expressing cells (IC50 = 445 nM)	[45]
TRPV1	Agonist	↑ [Ca^++^] levels in TRPV1-expressing HEK 293 cells (EC_50_ = 3.5 μM)	[46]
FAAH	Inhibitor	↓ [^14^C] AEA update in N18TG2 cells (IC_50_ = 27.5 µM) ↓ [^14^C] AEA update in HeLa cells expressing rat, but not human, FAAH at 50–200 μM	[46,47]
5-HT_1A_	Agonist	↓ [^3^H]8-OH-DPAT binding; ↑ GTPγS binding in 5-HT_1A_-expressing CHO cells	[48]
D2	Partial agonist	↓ [^3^H]domperidone binding to D2 receptors (Ki = 11 nM at D2_High_; Ki = 2800 nm at D2Low)	[45]
MOR & DOR	Allosteric modulator	↓ [^3^DNM] binding to MOR (IC_50_ = 7 μM) At 100 μM, ↑ [^3^H]DAMGO dissociation from MOR (pE_50_ = 4.38 μM) ↑ [^3^H]-naltrindole dissociation from DOR (pE_50_ = 4.1 μM)	[49,50]

CRE, cAMP response element; BRET, bioluminescence resonance energy transfer.

## Data Availability

Not applicable.
